# Visual Cortical Response Variability in Infants at High Familial Likelihood for Autism

**DOI:** 10.64898/2026.03.05.709374

**Published:** 2026-03-09

**Authors:** Abigail Dickinson, Madison Booth, Scott Huberty, Declan Ryan, Alana Campbell, Jessica B. Girault, Neely Miller, Bonnie Lau, John Zempel, Sara Jane Webb, Jed Elison, Adrian KC Lee, Annette Estes, Stephen R. Dager, Heather Hazlett, Jason Wolff, Robert Schultz, Natasha Marrus, Alan Evans, Joseph Piven, John R. Pruett, Shafali Jeste

**Affiliations:** a Center for Autism Research and Treatment, Semel Institute for Neuroscience, University of California, Los Angeles, CA, USA; b Department of Pediatrics, David Geffen School of Medicine, University of California, Los Angeles, CA, USA; c Carolina Institute for Developmental Disabilities, Department of Psychiatry, University of North Carolina at Chapel Hill, Chapel Hill, NC, USA; d Minnesota Center for Twin and Family Research, Department of Psychology, University of Minnesota, Minneapolis, MN, USA; e Department of Otolaryngology – Head and Neck Surgery, University of Washington, Seattle, WA, USA; f Department of Psychiatry, Washington University School of Medicine, St. Louis, MO, USA; g Center for Child Health, Behavior and Development, Seattle Children’s Research Institute, Seattle, WA, USA; h Institute of Child Development, Department of Pediatrics, University of Minnesota, Minneapolis, MN, USA; i Department of Speech and Hearing Sciences, Institute for Learning and Brain Sciences, University of Washington, Seattle, WA, USA; j Department of Radiology, University of Washington, Seattle, WA, USA; k Department of Educational Psychology, University of Minnesota, Minneapolis, MN, USA; l Center for Autism Research, Children’s Hospital of Philadelphia, University of Pennsylvania Perelman School of Medicine, Philadelphia, PA, USA; m McGill Centre for Integrative Neuroscience, Montreal Neurological Institute, McGill University, Montréal, QC, Canada

**Keywords:** Autism, EEG, Visual evoked potentials, infancy, development, neural variability

## Abstract

Visual processing undergoes rapid refinement in the first year of life, supporting the emergence of higher-order cognitive, language, and motor functions. Visual evoked potentials (VEPs) provide a non-invasive measure of visual system maturation that may shed light on heterogeneous developmental trajectories among infants at high familial likelihood for autism.

Infants with an older sibling with autism spectrum disorder (N = 177 at 6 months; N = 132 at 12 months) participated in the Infant Brain Imaging Study–Early Prediction (IBIS-EP) study. Pattern-reversal VEPs were recorded at 6 and 12 months, and developmental skills were assessed at 24 months using the Bayley Scales of Infant and Toddler Development (Bayley-III). VEP components were characterized by P1 amplitude, latency, and trial-to-trial variability in latency. Associations with 24-month cognitive, language, and motor scores were examined using general linear models controlling for age, site, sex, and trial count.

Robust VEPs were observed at both timepoints, showing age-appropriate morphology and expected developmental changes, including decreases in P1 latency and amplitude from 6 to 12 months. Greater trial-to-trial variability in P1 latency at both timepoints was significantly associated with higher cognitive and language scores at 24 months.

Variability in visual cortical response timing was the strongest neural correlate of developmental skills in infancy. These findings suggest that temporal variability in early neural responses may reflect adaptive sensory circuit flexibility rather than inefficiency, potentially facilitating experience-dependent tuning of visual pathways. VEPs offer a mechanistic window into how developing sensory systems scaffold individual differences in early developmental trajectories.

## 1. Introduction

Identification of early markers of atypical neurodevelopment, before clinical diagnoses such as autism can be made, is essential for understanding mechanisms that shape developmental variability and for informing earlier detection and intervention strategies. The development of basic sensory systems may be particularly valuable in this context, as sensory systems are among the first functional neural networks to mature, and their maturation provides the foundation for higher-order processes, including attention, language, and social cognition (Braddick & Atkinson, 2011; Chorna et al., 2024; Siu & Murphy, 2018). The visual system undergoes rapid structural and functional refinement during the first year of life, supporting improvements in acuity, depth perception, motion tracking, and attentional control (M. H. Johnson, 1990). This period of visual circuit maturation provides the scaffolding for complex behaviors such as joint attention, face and object recognition, and the integration of visual input with motor planning and early language learning (M. H. Johnson et al., 2015). Disruptions in these early visual processes may limit opportunities to coordinate perception and action or to map visual information onto emerging communication systems, leading to cascading effects on broader developmental outcomes.

Visual evoked potentials (VEPs) provide a non-invasive, temporally precise, and scalable method for probing early sensory-cortical mechanisms. Recorded using electroencephalography (EEG), VEPs capture the brain’s time-locked response to visual stimuli and index neural signaling within occipital cortex and associated visual pathways. The VEP is a stereotyped EEG waveform characterized by distinct components, including an early negative deflection (N1) followed by a positive peak (P1), which together reflect synaptic transmission and postsynaptic activity in primary and extra-striate visual areas (Creel, 2019). Variation in the latency and amplitude of these early components are thought to index the maturation of visual circuits, including increased myelination, synaptic efficiency, and the excitatory–inhibitory balance (Siu & Murphy, 2018). Trial-to-trial variability in response timing also offers complementary information about the stability and reliability of neural signaling, reflecting the consistency of cortical recruitment and the precision of sensory encoding (Milne, 2011). Together, these features make VEPs a powerful tool for quantifying early circuit function and its role in supporting infant development.

Beyond mean response properties, the trial-to-trial consistency of neural responses offers an additional window into circuit organization. Although variability in neural signals is sometimes treated as noise, converging evidence suggests that during early sensitive periods, greater variability may reflect a system that remains open to experience-dependent tuning, a property that may be functionally beneficial rather than detrimental (Deguire et al., 2023; Faisal et al., 2008). Accumulating evidence links individual differences in these neural visual responses to variation in developmental outcomes. For instance, P1 and N1 latencies have been associated with motor skills in young children (Otten et al., 2025) and with cognitive and psychomotor scores in infants with suspected developmental delays (Kim et al., 2018). Prospective longitudinal studies further indicate that early visual response properties are related to later developmental trajectories. For example, P1 latency at 3 months predicted cognitive scores at 18 months in infants exposed to gestational diabetes (Torres-Espínola et al., 2018), and P1 amplitude at 6 months was associated with motor, cognitive, and language outcomes at 27 months in children exposed to early adversity (Jensen et al., 2019). These findings suggest that VEP measures of visual circuit function offer a promising approach for capturing early variability in neural circuit development that is relevant to understanding later developmental outcomes.

While VEP metrics have shown promise for elucidating individual differences in early neural processing and later developmental outcomes in other at-risk populations (e.g., (Jensen et al., 2019; Torres-Espínola et al., 2018)), it remains unclear whether similar associations are present in infants at high familial likelihood (HL) for autism. HL infants, defined by having an older sibling with autism, have elevated rates of autism recurrence (~20%; (Ozonoff et al., 2011, 2024)) and higher rates (~30%) of broader neurodevelopmental delays (Charman et al., 2017; Messinger et al., 2013). As such, this group provides a unique opportunity to study mechanisms of atypical brain development, as they can be identified from birth, exhibit wide variability in developmental trajectories, and are at increased likelihood for atypical cognitive, language, and motor outcomes. Previous EEG research in HL infants has shown that early oscillatory activity is linked to later development, including associations between resting alpha power at 3 months and language outcomes at 12 months (Levin et al., 2017). However, it remains unclear whether evoked responses such as VEPs capture variability in early sensory circuit maturation relevant to later developmental outcomes in this population.

In the present Infant Brain Imaging (IBIS) study, we examined whether individual differences in early visual cortical processing relate to later developmental outcomes within HL infants. Specifically, we aimed to test whether variations in early neural circuit function (quantified through VEP indices of P1 amplitude, P1 latency, and trial-to-trial latency variability) map onto cognitive, language, and motor abilities at 24 months. This approach allows us to assess how early differences in the efficiency and stability of sensory processing, reflected in both the timing and consistency of visual cortical responses, contribute to emerging developmental trajectories. We hypothesized that shorter P1 latencies would be associated with stronger developmental outcomes at 24 months, consistent with latency as an index of visual system maturation. We additionally examined trial-to-trial variability in P1 latency as a complementary index of neural circuit flexibility, given emerging evidence that response variability during early sensitive periods may reflect adaptive plasticity. The large multi-site longitudinal cohort design facilitated a robust sample size with rigorous and standardized EEG and behavioral data collection across five national sites. It should be noted that the present report focuses on continuous developmental outcomes, and analyses examining EEG measures as predictors of categorical autism diagnostic outcomes are planned once the full cohort has been accrued in accordance with pre-specified statistical power targets. This consortium has been investigating neural and behavioral markers of atypical development in infants with and without familial likelihood for autism for more than a decade.

## 2. Methods

### 2.1. Participants

Participants were part of the Infant Brain Imaging Study-Early Prediction (IBIS-EP) study, a prospective cohort study of infant siblings of children with autism, across five sites: Washington University in St. Louis, University of Washington in Seattle, Children’s Hospital of Philadelphia, University of Minnesota, and the University of North Carolina at Chapel Hill. All infants had an older full sibling with a confirmed diagnosis of autism spectrum disorder (ASD), validated via medical records, the Social Communication Questionnaire (SCQ; (Rutter, Bailey, et al., 2003)), and the Autism Diagnostic Interview–Revised (ADI-R; (Rutter, Le Couteur, et al., 2003)).

Eligibility criteria included: (1) gestational age > 36 weeks; (2) absence of medical or neurological conditions affecting growth, development, or cognition (e.g., seizure disorders) or significant sensory impairments (e.g., vision or hearing loss); (3) no known genetic syndromes associated with ASD; (4) no immediate family history of psychosis, schizophrenia, or bipolar disorder; (5) no contraindications for MRI; and (6) English as the primary language in the home. These criteria were assessed during a structured family history interview and are consistent with prior IBIS protocols (Emerson et al., 2017; Hazlett et al., 2017). All procedures were approved by a centralized institutional review board at Washington University in St. Louis. Written informed consent was obtained from a parent or guardian for each participant, in accordance with the Declaration of Helsinki.

EEG data were collected at 6 and 12 months of age, and behavioral assessments were completed at 24 months. The present analyses focused on participants with EEG at 6 and/or 12 months and available behavioral data at 24 months. Data reflect a cutoff as of January 2025, with data collection continuing until early 2027. VEP data were collected from 180 infants at 6 months and 135 infants at 12 months. Of these infants, VEP data were useable for 177/180 of the 6-month data, and 132/135 of 12-month data. Behavioral outcome data at 24 months were available for 98 and 97 of those infants, respectively. Demographic characteristics of the final analytic sample are presented in Table 1.

### 2.2. Behavioral Assessments

At 24 months, all participants completed the Bayley Scales of Infant and Toddler Development, Third Edition (Bayley-III) (Bayley, 2006), a standardized measure of early developmental functioning. The assessment was administered by a licensed clinical psychologist or trained developmental specialist, following standardized administration procedures. Age-corrected composite scores were derived for three core domains: Cognitive, Language (combined expressive and receptive), and Motor (combined fine and gross). Scores were based on each child’s chronological age at the time of assessment, in line with Bayley-III scoring guidelines.

### 2.3. EEG Collection

Visual evoked potentials (VEPs) were collected as part of a broader EEG battery that included resting-state and auditory paradigms (for full details see (Dickinson et al., 2024)). Acquisition procedures were standardized across IBIS-EP sites. Infants were seated on their caregiver’s lap in a dimly lit room, 57 cm from a laptop screen. A black-and-white checkerboard stimulus (36 × 36 checks; 21 × 21 cm) with a central red fixation point underwent phase reversals every 500ms for 160 trials. The checkerboard had 99% contrast, an average luminance of 80 cd/m^2^, and subtended approximately 21° of visual angle. Stimulus presentation was controlled by E-Prime (Psychology Software Tools), and phase reversals were marked using a screen-mounted photocell connected to the EEG amplifier. EEG was recorded using Net Amps amplifiers and high-density HydroCel Geodesic Sensor Nets (EGI) at a sampling rate of 500–1000 Hz (see (Dickinson et al., 2024) for site-specific EEG collection details). Attention was monitored and documented by an experimenter throughout the session.

### 2.4. EEG Processing

EEG data were processed using EEGLAB (Delorme & Makeig, 2004) and custom MATLAB scripts (The MathWorks, Inc., Natick, MA). Continuous data were band-pass filtered (0.3–30 Hz, FIR) and any periods of inattention noted by the examiner were excluded. Data were then visually inspected to remove artifactual sections of data and channels. After removing artifacts, all datasets were interpolated to a standard 33-channel 10–10 montage (including O1, O2, Oz) and re-referenced to the average of all channels. Data were epoched −100 to 300 ms around stimulus onset (defined by photocell triggers) and baseline-corrected (−100 to 0 ms).

Analyses were restricted to conventional VEP electrode sites (O1, O2, Oz) overlying the occipital cortex, where visual responses are typically strongest (e.g., (Kovarski et al., 2019)). Trials were screened for artifacts, and any trials containing eye movements, blinks, or voltages exceeding ±150 μV were excluded. Trials without a characteristic VEP morphology were removed. Specifically, trials were only retained if they exhibited a positive P1 peak (70–150 ms) preceded by a negative deflection (N1), with a monotonic rise between N1 and P1 components. See Supplementary Figure 1 for examples of accepted and rejected trial morphologies. Following artifact and morphology-based exclusions, participants contributed an average of 48.57 (SD = 17.72; Range = 15–98) artifact-free trials at 6 months and 42.24 (SD = 13.93; Range =19–97) at 12 months. The number of useable trials was used as a covariate in all analyses, as described below.

For each occipital channel, quality-controlled trials were averaged to yield a mean VEP. From this waveform, we extracted P1 latency and amplitude, characterized by the N1–P1 amplitude difference. Trial-level P1 latencies were used to compute intra-individual variability, indexed by the median absolute deviation (MAD) across trials (Kovarski et al., 2019; Milne, 2011). Final EEG metrics (P1 latency, amplitude, and variability) were averaged across occipital channels.

### 2.5. Statistical Analyses

Statistical analyses were conducted in MATLAB (The MathWorks, Natick, MA) and R. Three separate linear mixed-effects models were used to characterize developmental changes in P1 latency, P1 amplitude, and P1 latency variability between 6 and 12 months. Participant was included as a random effect to account for repeated measurements in participants with data at both timepoints, and site, sex, and the number of artifact-free trials were included as fixed-effect covariates.

Separate general linear models were used to examine associations between VEP metrics measured at 6 and 12 months and cognitive, language, and motor outcomes assessed at 24 months. For each EEG timepoint (6 and 12 months), nine models were fit (3 developmental outcomes × 3 EEG predictors: P1 amplitude, P1 latency, and P1 latency variability). All models included age at EEG acquisition (in months), site (five-level categorical variable), sex, and number of artifact-free trials as covariates. Because P1 amplitude and latency variability are strongly related, models examining P1 amplitude additionally controlled for latency variability, and models examining latency variability controlled for amplitude. False discovery rate (FDR) correction using the Benjamini–Hochberg procedure was applied separately for the six-month and twelve-month model families to adjust for multiple comparisons. Table 2 reports regression coefficients and two-sided *p* values for the EEG predictors of interest, with associations surviving FDR correction (*q* < .05) indicated in bold. Full regression outputs for all models, including coefficients for covariates, are provided in Supplementary Tables S1–S2.

## 3. Results

Linear mixed-effects models accounting for repeated measures, site, sex, and trial count indicated significant developmental changes in all three EEG metrics from 6 to 12 months. Average P1 latency decreased significantly from 109.38 ms (SD = 10.07) at 6 months to 105.41 ms (SD = 8.49) at 12 months, β = 4.22, SE = 1.19, t(187) = 3.54, p < .001. P1 amplitude was also significantly greater at 6 months (M = 15.28 μV, SD = 4.66) than at 12 months (M = 13.15 μV, SD = 4.44), β = 1.83, SE = 0.53, t(187) = 3.47, p < .001. In contrast, trial-to-trial variability in P1 latency increased from 6 months (M = 15.59 ms, SD = 3.22) to 12 months (M = 16.89 ms, SD = 2.41), β = −0.87, SE = 0.32, t(187) = −2.70, p = .008.

**Figure 3.**
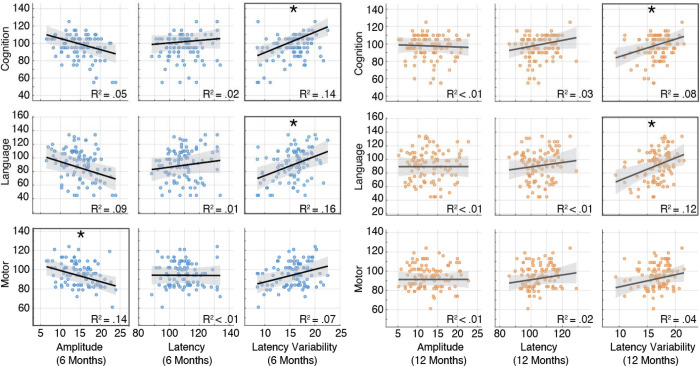
Scatterplots illustrating associations between EEG metrics (P1 latency, P1 latency variability, and P1 amplitude) measured at 6 months (blue) and 12 months (orange) and developmental outcomes at 24 months. *R*^2^ values reflect bivariate associations. Associations that remained significant following FDR correction (*q* < .05) are indicated with an asterisk.

Associations between P1 amplitude, latency, and latency variability and developmental outcomes at 24 months were examined (Table 2; Figure 3). These analyses revealed a consistent pattern of associations between trial-to-trial variability in P1 latency and later developmental outcomes. While mean P1 latency at either 6 or 12 months did not show significant associations with any developmental outcomes, greater P1 latency variability at 6 months was associated with higher cognitive (β = 1.85, *p* = .005) and language composite scores (β = 2.10, *p* = .016) at 24 months. Similar associations were observed at 12 months, such that greater P1 latency variability was associated with higher cognitive (β = 1.95, *p* = .007) and language (β = 2.87, *p* = .007) scores. These associations were observed after controlling for the number of artifact-free trials, indicating that greater variability reflects a neural signal property rather than an artifact of trial count. All associations between P1 latency variability and cognitive and language outcomes remained significant following correction for multiple comparisons (Table 2). A significant association between P1 latency variability at 12 months and motor outcomes at 24 months was also observed; however, this association did not survive correction for multiple comparisons.

P1 amplitude showed a limited and isolated association with developmental outcomes. Although greater P1 amplitude at 6 months was significantly associated with motor outcomes at 24 months (β = −0.92, *p* = .002), it was not associated with cognitive or language outcomes. Similarly, P1 amplitude measured at 12 months was not significantly associated with any developmental outcome. Detailed model estimates for each outcome and EEG metric are reported in Supplementary Tables S1 (6-month EEG) and S2 (12-month EEG).

## 4. Discussion

In the present study, we examined the association between visual cortical processing and developmental outcomes in infants at high familial likelihood for autism. Using VEPs recorded at 6 and 12 months, we found that P1 latency variability (reflecting trial-to-trial consistency in neural response timing) was significantly associated with cognitive and language skills at 24 months. Infants who exhibited greater variability in the timing of their visual cortical responses showed higher cognitive and language scores at 24 months. In contrast, average P1 latency was not associated with developmental outcomes, and P1 amplitude (at 6 months) showed a single, isolated association with later motor skills. Together, these findings suggest that variability in early visual cortical responses may be a particularly informative physiological marker of emerging cognitive and language abilities in HL infants.

Across the cohort, we observed well-defined VEPs at both 6 and 12 months, characterized by age-appropriate morphology and expected developmental changes. Specifically, P1 latency and amplitude decreased from 6 to 12 months, consistent with ongoing myelination and increasing synaptic efficiency within visual pathways during the first year of life (Kovarski et al., 2019; MCculloch, 2013; Skoczenski & Norcia, 2002). These normative developmental shifts indicate that the VEP measures used here are sensitive to typical visual system maturation in infancy. Despite these clear developmental changes, mean P1 latency did not relate to later cognitive, language, or motor outcomes. While some prior studies have reported associations between infant VEP latency and later developmental skills (e.g., (Otten et al., 2025; Torres-Espínola et al., 2018), others have not (Jensen et al., 2019). This inconsistency suggests that between-participant averages of latency may represent a relatively coarse index of neural development, potentially obscuring functionally meaningful within-participant dynamics.

In contrast, trial-to-trial variability in P1 latency emerged as a consistently developmentally meaningful signal. Neural response variability is a defining feature of early development. Newborns show substantially greater intra-individual variability in VEP responses than older infants (Benavente et al., 2005), and neural variability more broadly is highest early in life before declining across maturation (Hultsch et al., 2011). Our findings indicate that, during the first year of life, greater variability in the timing of visual cortical responses predicts more favorable cognitive and language outcomes, suggesting that higher neural response variability at this stage may be adaptive.

Specifically, higher neural response variability may reflect more flexible and dynamically organizing sensory circuits. Such flexibility may allow visual pathways to remain highly responsive to experience during a critical period of learning, supporting downstream cognitive and language development, in line with the fact that early visual development is known to support communication, motor planning, and social interaction (Braddick & Atkinson, 2011; Chorna et al., 2024; Siu & Murphy, 2018). In this context, greater temporal variability in visual cortical responses may index a system that is still actively calibrating sensory predictions and integrating experience, rather than one that has prematurely stabilized. This interpretation aligns with cascading models of neurodevelopment, in which early sensory circuit organization provides the neural scaffolding for the emergence of higher-order cognitive, language, and social functions (S. P. Johnson, 2011; Karmiloff-Smith, 1998), a framework that has also been proposed as central to the developmental underpinnings of autism (Girault, 2025; Piven et al., 2017).

The notion that elevated neural variability during early development can be adaptive is supported by converging evidence from other modalities and age groups. BOLD fMRI studies have shown that greater neural signal variability is associated with flexible and optimal brain function (Armbruster-Genç et al., 2016; Nomi et al., 2017). Similarly, recent EEG work has demonstrated that infants who show more differentiated or variable neural responses to changes in sensory input during the first year of life exhibit better adaptive functioning at age four (Deguire et al., 2023). Together with the present findings, this body of work supports the view that elevated neural variability early in development reflects prolonged plasticity and exploratory circuit tuning that benefits learning. This interpretation aligns closely with prior structural MRI findings (in two separate infant samples, including a prior IBIS cohort) showing that infants who exhibit more protracted or dynamically changing white-matter development, particularly in pathways supporting visual and associative processing, demonstrate stronger later cognitive and language outcomes (Girault et al., 2022; Swanson et al., 2017). Taken together, these structural and functional findings suggest that early developmental advantage may arise not from rapid stabilization of neural systems, but from extended windows of plasticity that allow circuits to be shaped by experience. A critical next step will be to examine white-matter development in the same infants studied here to determine whether greater inter-trial variability in visual cortical responses is associated with more gradual or protracted white-matter maturation.

It is also important to discuss our findings in the context of prior work showing elevated neural response variability later in development in autism. EEG studies in older children and adults with autism have consistently reported increased intra-individual trial-to-trial variability in evoked responses, including greater variability in P1 amplitude and latency (Dinstein et al., 2012; Dong et al., 2024; Milne, 2011). Heightened variability in these older populations has often been interpreted as reflecting unreliable or inefficient neural signaling associated with perceptual and cognitive difficulties (Dinstein et al., 2012; Milne, 2011). This apparent discrepancy underscores the importance of developmental context. Neural variability may serve different functional roles at different stages of development, supporting adaptive flexibility during early sensitive periods, but reflecting dysregulated or unstable processing later in development. Longitudinal studies will be essential for determining how early-life neural response variability relates to autism diagnosis and for understanding how variability in infancy maps onto neural response patterns observed in later childhood.

Several limitations should be considered. Although this study included a large, well-characterized cohort, analyses were limited to infants at high familial likelihood for autism. While this focus is important for understanding early neural markers that may elucidate later developmental variability within this highly heterogeneous group, future work should examine HL infants alongside infants from the general population to determine whether the observed associations generalize across likelihood groups or reflect patterns specific to familial risk. In addition, the present analyses were designed to characterize continuous variation in developmental outcomes within the HL cohort rather than categorical diagnostic endpoints. Future analyses will test whether early differences in sensory-cortical processing are also associated with later categorical diagnostic outcomes once the full sample, consistent with pre-specified power targets, is available.

In summary, this study demonstrates that variability in the timing of early visual cortical responses is a robust predictor of later cognitive and language skills in infants at elevated familial likelihood for autism. These findings suggest that both the efficiency and flexibility of early sensory processing contribute to individual differences in developmental trajectories. By quantifying neural timing and consistency during the first year of life, VEP measures provide a mechanistic window into how early sensory systems support the emergence of higher-order developmental skills. More broadly, this work highlights sensory-cortical flexibility as a key mechanism shaping early development and underscores the potential of EEG-based biomarkers to inform early surveillance and intervention strategies in infants at elevated neurodevelopmental risk.

## Supplementary Material

Supplement 1

Supplement 2

Supplement 3

## Figures and Tables

**Figure 1.**
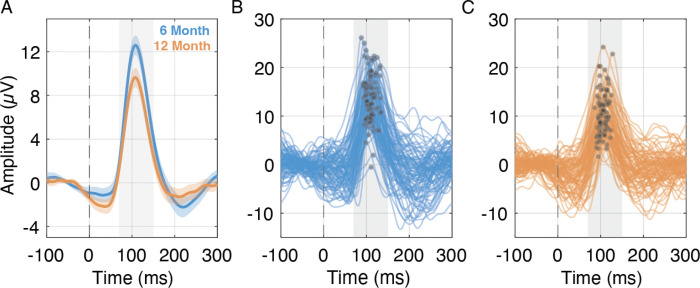
A) Grand average pattern-reversal visual evoked potentials (VEPs) at 6 months (blue) and 12 months (orange). Solid lines represent the mean VEP waveform across participants; shaded regions depict the 95% confidence interval. B–C) Individual VEP waveforms from participants at 6 months (B) and 12 months (C). Each line reflects the average waveform for a single participant. P1 components are marked with filled circles within the typical P1 latency window.

**Figure 2.**
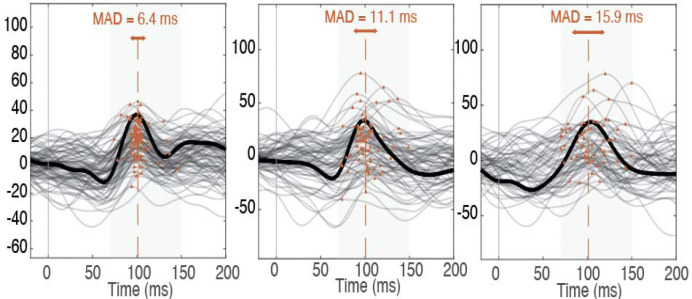
Examples of trial-level P1 identification in individual participants, illustrating variability in P1 latency. Intra-individual variability in latency was quantified as the median absolute deviation (MAD) of P1 latency (marked in red dots) across individual trials. Each subplot (A–C) shows a different participant, selected to represent the low (A), middle (B), and high (C) trial-to-trial P1 latency variability (MAD). Importantly, while average waveforms may appear similar, underlying variability in latency differs substantially.

**Table 1.** Participant Demographics.

	6 Month	12 Month

EEG Recorded, *n*	180	135
EEG Useable, *n*	177	132
Also Have 24 Month Behavior, *n*	98	97
Age of EEG (months; *M (SD))*	6.79 (0.80)	12.72 (0.71)
Sex *(*female*; n* (%))	53 (54.1%)	49 (50.5%)
Race, *n* (%)		
Asian	5 (5.1%)	1 (1.0%)
Black	6 (6.1%)	7 (7.2%)
Hispanic	5 (5.1%)	5 (5.2%)
More than one race	12 (12.2%)	8 (8.2%)
White	70 (71.4%)	76 (78.4%)
Ethnicity, *n* (%)		
Hispanic	24 (24.5%)	26 (26.8%)
Non-Hispanic	74 (75.5%)	71 (73.2%)
Household Income, *n* (%)		
<50K	26 (26.5%)	27 (27.8%)
50K-150K	44 (44.9%)	42 (43.3%)
>150K	23 (23.5%)	25 (25.8%)
Unknown	5 (5.1%)	3 (3.1%)
Maternal Education, *n* (%)		
< Some College	11 (11.2%)	11 (11.3%)
> Some College	55 (56.1%)	53 (54.6%)
Graduate Training	31 (31.6%)	32 (33.0%)
Unknown	1 (1%)	1 (1.0%)
VEP Metrics*, M (SD)*		
P1 Amplitude (μV)	15.28 (4.66)	13.15 (4.44)
P1 Latency (ms)	109.42 (10.02)	105.41 (8.49)
P1 Latency Variability (ms)	15.59 (3.22)	16.89 (2.41)

*Note.* Participant counts are not mutually exclusive. Some infants contributed EEG data at both 6 and 12 months. Specifically, 68 participants provided EEG data at both time points and had 24-month behavioral data. These participants are included in both columns.

**Table 2.** Associations between EEG metrics and developmental outcomes at 24 months. Values represent regression coefficients (β) and corresponding *p* values for the EEG predictors of interest from each model. Models were estimated separately for each EEG metric and outcome domain. Significant associations that survived false discovery rate (FDR) correction (*q* < .05) are bolded and marked with an asterisk. Full model results, including coefficients for all covariates, are provided in Supplementary Tables 1–2.

	24 Month Behavioral Composite Scores		
VEP Metric	Cognitive	Language	Motor
	*β (p; q)*	*β (p; q)*	*β (p; q)*

*6 Months*			
P1 Amplitude (μV)	−0.36 (.284; .320)	−0.97 (.058; .131)	−0.94 (.001; .005)*
P1 Latency (ms)	0.20 (.230; .296)	0.32 (.206; .296)	0.01 (.949; .949)
P1 Latency Variability (ms)	1.85 (.001; .005)*	2.10 (.016; .048)*	0.65 (.174; .296)
*12 Months*			
P1 Amplitude (μV)	−0.15 (.653; .840)	0.15 (.766; .862)	0.01 (.961; .961)
P1 Latency (ms)	0.33 (.085; .191)	0.32 (.263; .395)	0.25 (.138; .248)
P1 Latency Variability (ms)	1.95 (.007; .032)*	2.87 (.007; .032)*	1.24 (.049; .147)

## Research Highlights

- Trial-to-trial variability in visual cortical response timing predicts cognitive and language outcomes at 24 months in infants at familial likelihood for autism.
- Mean P1 latency did not predict outcomes, suggesting variability is a more sensitive early neural marker than average response timing.
- Greater neural response variability in infancy may reflect adaptive sensory circuit flexibility rather than noise or inefficient processing.
- VEP-based biomarkers provide a scalable mechanistic window into how early sensory processing scaffolds cognitive and language development.

## References

[R1] Armbruster-Genç D. J., Ueltzhöffer K., & Fiebach C. J. (2016). Brain signal variability differentially affects cognitive flexibility and cognitive stability. Journal of Neuroscience, 36(14), 3978–3987. 27053205 10.1523/JNEUROSCI.2517-14.2016 PMC6705511

[R2] Bayley N. (2006). Bayley scales of infant and toddler development.

[R3] Benavente I., Tamargo P., Tajada N., Yuste V., & Oliván M. J. (2005). Flash visually evoked potentials in the newborn and their maturation during the first six months of life. Documenta Ophthalmologica, 110(2), 255–263. 16328934 10.1007/s10633-005-0818-0

[R4] Braddick O., & Atkinson J. (2011). Development of human visual function. Vision Research, 51(13), 1588–1609. 21356229 10.1016/j.visres.2011.02.018

[R5] Charman T., Young G. S., Brian J., Carter A., Carver L. J., Chawarska K., Curtin S., Dobkins K., Elsabbagh M., Georgiades S., Hertz-Picciotto I., Hutman T., Iverson J. M., Jones E. J., Landa R., Macari S., Messinger D. S., Nelson C. A., Ozonoff S., … Zwaigenbaum L. (2017). Non-ASD outcomes at 36 months in siblings at familial risk for autism spectrum disorder (ASD): A baby siblings research consortium (BSRC) study. Autism Research, 10(1), 169–178. 10.1002/aur.1669 27417857 PMC5993543

[R6] Chorna O., Corsi G., Del Secco S., Bancale A., & Guzzetta A. (2024). Correlation between early visual functions and cognitive outcome in infants at risk for cerebral palsy or other neurodevelopmental disorders: A systematic review. Children, 11(6), 747. 38929326 10.3390/children11060747 PMC11201713

[R7] Creel D. J. (2019). Visually evoked potentials. Handbook of Clinical Neurology, 160, 501–522. 31277872 10.1016/B978-0-444-64032-1.00034-5

[R8] Deguire F., Lopez-Arango G., Knoth I. S., Côté V., Agbogba K., & Lippé S. (2023). EEG repetition and change detection responses in infancy predict adaptive functioning in preschool age: A longitudinal study. Scientific Reports, 13(1), 9980. 37340003 10.1038/s41598-023-34669-9 PMC10282122

[R9] Delorme A., & Makeig S. (2004). EEGLAB: an open source toolbox for analysis of single-trial EEG dynamics including independent component analysis. Journal of Neuroscience Methods, 134, 9–21. 10.1016/j.jneumeth.2003.10.009 15102499

[R10] Dickinson A., Booth M., Daniel M., Campbell A., Miller N., Lau B., Zempel J., Webb S. J., Elison J., & Lee A. K. (2024). Multi-site EEG studies in early infancy: Methods to enhance data quality. Developmental Cognitive Neuroscience, 69, 101425. 10.1016/j.dcn.2024.101425 PMC11380169 39163782

[R11] Dinstein I., Heeger D. J., Lorenzi L., Minshew N. J., Malach R., & Behrmann M. (2012). Unreliable evoked responses in autism. Neuron, 75(6), 981–991. 22998867 10.1016/j.neuron.2012.07.026 PMC3457023

[R12] Dong M., Telesca D., Guindani M., Sugar C., Webb S. J., Jeste S., Dickinson A., Levin A. R., Shic F., & Naples A. (2024). Modeling intra individual inter trial EEG response variability in autism. Statistics in Medicine, 43(17), 3239–3263. 38822707 10.1002/sim.10131 PMC12096858

[R13] Emerson R. W., Adams C., & Nishino T. (2017). Functional neuroimaging of high-risk 6-month-old infants predicts a diagnosis of autism at 24 months of age. Science. http://stm.sciencemag.org/content/9/393/eaag2882.abstract 10.1126/scitranslmed.aag2882 PMC5819345 28592562

[R14] Faisal A. A., Selen L. P., & Wolpert D. M. (2008). Noise in the nervous system. Nature Reviews Neuroscience, 9(4), 292–303. 18319728 10.1038/nrn2258 PMC2631351

[R15] Girault J. B. (2025). The developing visual system: A building block on the path to autism. Developmental Cognitive Neuroscience, 73, 101547. 10.1016/j.dcn.2025.101547 PMC11964655 40096794

[R16] Girault J. B., Donovan K., Hawks Z., Talovic M., Forsen E., Elison J. T., Shen M. D., Swanson M. R., Wolff J. J., & Kim S. H. (2022). Infant visual brain development and inherited genetic liability in autism. American Journal of Psychiatry, 179(8), 573–585. 35615814 10.1176/appi.ajp.21101002 PMC9356977

[R17] Hazlett H. C., Gu H., Munsell B. C., Kim S. H., Styner M., Wolff J. J., Elison J. T., Swanson M. R., Zhu H., & Botteron K. N. (2017). Early brain development in infants at high risk for autism spectrum disorder. Nature, 542(7641), 348–351. 28202961 10.1038/nature21369 PMC5336143

[R18] Hultsch D. F., Strauss E., Hunter M. A., & MacDonald S. W. (2011). Intraindividual variability, cognition, and aging. In The handbook of aging and cognition (pp. 491–556). Psychology Press.

[R19] Jensen S. K., Kumar S., Xie W., Tofail F., Haque R., Petri W. A., & Nelson C. A. (2019). Neural correlates of early adversity among Bangladeshi infants. Scientific Reports, 9(1), 3507. 30837491 10.1038/s41598-019-39242-x PMC6401115

[R20] Johnson M. H. (1990). Cortical maturation and the development of visual attention in early infancy. Journal of Cognitive Neuroscience, 2(2), 81–95. 23972019 10.1162/jocn.1990.2.2.81

[R21] Johnson M. H., Gliga T., Jones E., & Charman T. (2015). Annual Research Review: Infant development, autism, and ADHD–early pathways to emerging disorders. Journal of Child Psychology and Psychiatry, 56(3), 228–247. 25266278 10.1111/jcpp.12328

[R22] Johnson S. P. (2011). Development of visual perception. Wiley Interdisciplinary Reviews. Cognitive Science, 2(5), 515–528. 10.1002/wcs.128 26302303

[R23] Karmiloff-Smith A. (1998). Development itself is the key to understanding developmental disorders. Trends in Cognitive Sciences, 2(10), 389–398. 21227254 10.1016/s1364-6613(98)01230-3

[R24] Kim J., Sung I. Y., Ko E. J., & Jung M. (2018). Visual evoked potential in children with developmental disorders: Correlation with neurodevelopmental outcomes. Annals of Rehabilitation Medicine, 42(2), 305. 29765884 10.5535/arm.2018.42.2.305 PMC5940607

[R25] Kovarski K., Malvy J., Khanna R. K., Arsène S., Batty M., & Latinus M. (2019). Reduced visual evoked potential amplitude in autism spectrum disorder, a variability effect? Translational Psychiatry, 9(1), 341. 31852886 10.1038/s41398-019-0672-6 PMC6920480

[R26] Levin A. R., Varcin K. J., O’Leary H. M., Tager-Flusberg H., & Nelson C. A. (2017). EEG power at 3 months in infants at high familial risk for autism. Journal of Neurodevelopmental Disorders, 9. 10.1186/s11689-017-9214-9 PMC5598007 28903722

[R27] MCculloch D. L. (2013). Visual evoked potentials in infants. In Infant EEG and event-related potentials (pp. 39–76). Psychology Press.

[R28] Messinger D., Young G. S., Ozonoff S., Dobkins K., Carter A., Zwaigenbaum L., Landa R. J., Charman T., Stone W. L., Constantino J. N., Hutman T., Carver L. J., Bryson S., Iverson J. M., Strauss M. S., Rogers S. J., & Sigman M. (2013). Beyond Autism: A Baby Siblings Research Consortium Study of High-Risk Children at Three Years of Age. Journal of the American Academy of Child & Adolescent Psychiatry, 52(3), 300–308.e1. 10.1016/j.jaac.2012.12.011 23452686 PMC3625370

[R29] Milne E. (2011). Increased intra-participant variability in children with autistic spectrum disorders: Evidence from single-trial analysis of evoked EEG. Frontiers in Psychology, 2. 10.3389/fpsyg.2011.00051 PMC3110871 21716921

[R30] Nomi J. S., Bolt T. S., Ezie C. C., Uddin L. Q., & Heller A. S. (2017). Moment-to-moment BOLD signal variability reflects regional changes in neural flexibility across the lifespan. Journal of Neuroscience, 37(22), 5539–5548. 28473644 10.1523/JNEUROSCI.3408-16.2017 PMC5452342

[R31] Otten K., Edgar J. C., Green H. L., Mol K., McNamee M., Kuschner E. S., Kim M., Liu S., Huang H., & Nordt M. (2025). The maturation of infant and toddler visual cortex neural activity and associations with fine motor performance. Developmental Cognitive Neuroscience, 71, 101501. 10.1016/j.dcn.2024.101501 PMC11743914 39733499

[R32] Ozonoff S., Young G. S., Bradshaw J., Charman T., Chawarska K., Iverson J. M., Klaiman C., Landa R. J., McDonald N., Messinger D., Schmidt R. J., Wilkinson C. L., & Zwaigenbaum L. (2024). Familial Recurrence of Autism: Updates From the Baby Siblings Research Consortium. Pediatrics, 154(2), e2023065297. 10.1542/peds.2023-065297 PMC11291960 39011552

[R33] Ozonoff S., Young G. S., Carter A., Messinger D., Yirmiya N., Zwaigenbaum L., Bryson S., Carver L. J., Constantino J. N., Dobkins K., Hutman T., Iverson J. M., Landa R., Rogers S. J., Sigman M., & Stone W. L. (2011). Recurrence Risk for Autism Spectrum Disorders: A Baby Siblings Research Consortium Study. Pediatrics, peds.2010–2825. 10.1542/peds.2010-2825 PMC3164092 21844053

[R34] Piven J., Elison J. T., & Zylka M. J. (2017). Toward a conceptual framework for early brain and behavior development in autism. Molecular Psychiatry, 22(10), 1385–1394. 10.1038/mp.2017.131 28937691 PMC5621737

[R35] Rutter M., Bailey A., & Lord C. (2003). SCQ. The Social Communication Questionnaire. Torrance, CA: Western Psychological Services.

[R36] Rutter M., Le Couteur A., & Lord C. (2003). Adi-r. Autism Diagnostic Interview Revised. Manual. Los Angeles: Western Psychological Services.

[R37] Siu C. R., & Murphy K. M. (2018). The development of human visual cortex and clinical implications. Eye and Brain, 25–36. 29760575 10.2147/EB.S130893 PMC5937627

[R38] Skoczenski A. M., & Norcia A. M. (2002). Late maturation of visual hyperacuity. Psychological Science, 13(6), 537–541. 12430838 10.1111/1467-9280.00494

[R39] Swanson M. R., Wolff J. J., Elison J. T., Gu H., Hazlett H. C., Botteron K., Styner M., Paterson S., Gerig G., & Constantino J. (2017). Splenium development and early spoken language in human infants. Developmental Science, 20(2), e12360. 10.1111/desc.12360 PMC4840090 26490257

[R40] Torres-Espínola F. J., Berglund S. K., García S., Pérez-García M., Catena A., Rueda R., Sáez J. A., Campoy C., & team P. (2018). Visual evoked potentials in offspring born to mothers with overweight, obesity and gestational diabetes. PloS One, 13(9), e0203754. 10.1371/journal.pone.0203754 PMC6135499 30208080

