# Supplementary material for: Visual Cortical Response Variability in Infants at High Familial Likelihood for Autism
Source: bioRxiv. 2026 Mar 9:2026.03.05.709374. Preprint. [Version 1] doi: 10.64898/2026.03.05.709374 (PMC13060789; doi:10.64898/2026.03.05.709374)
Supplement: Supplement 1 [file media-1.docx]

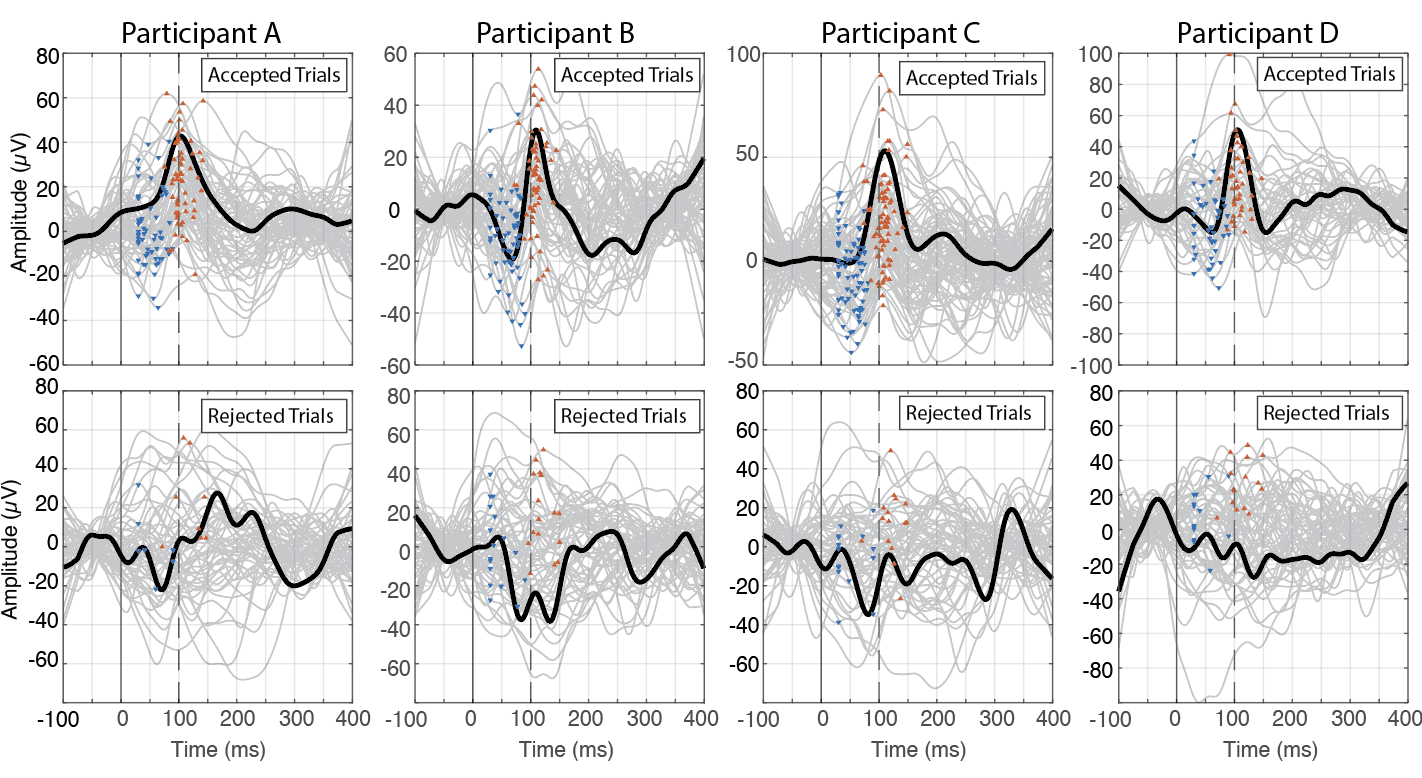
**Supplementary Figure 1.** Trial-level inclusion criteria and examples of retained versus excluded VEP trials. Trials were retained only if the peak amplitude did not exceed ±150 µV and the waveform exhibited a canonical N1–P1 morphology, which was defined as a positive deflection (P1; 70–150 ms) preceded by a negative deflection (N1), with a monotonic rise between the two. Panels A–D show four representative participants. Upper panels display retained trials (gray lines) with identified P1 (red triangles) and N1 (blue triangles); the black line indicates the average across trials. Lower panels show excluded trials that were rejected due to excessive amplitude, absent or mis-ordered peaks, or atypical morphology.
